# Supplementary material for: The Antimicrobial, Antioxidant, and Anticancer Activity of Greenly Synthesized Selenium and Zinc Composite Nanoparticles Using Ephedra aphylla Extract
Source: Biomolecules. 2021 Mar 22;11(3):470. doi: 10.3390/biom11030470 (PMC8005055; doi:10.3390/biom11030470)
Supplement: Supplementary file 1 [file biomolecules-11-00470-s001.pdf]

Supplementary file

The antimicrobial, antioxidant, and anticancer activity of greenly synthesized selenium and zinc composite nanoparticles using *Ephedra aphylla* extract

Mustafa Mohsen El Zayat[1], Mostafa M. Eraqi[2,3], Hani Alrefai[4,5*], Ayman Y. El-Khateeb[6], Marwan A. Ibrahim [3,7*], Hashim M. Aljohani[8,9], Maher M. Aljohani[10,11], Moustafa Mohammed Elshaer[12]

1. Unit of Genetic Engineering and Biotechnology, Faculty of Science, Mansoura University, Mansoura, Egypt
2. Dep. Of Microbiology and Immunology, Veterinary Research Division, National Research Center, Dokki 12622 Giza, Egypt.
3. Dep. of Biology, College of Science, Majmaah University, Majmaah 11952, Saudi Arabia
4. Medical Biochemistry Department, Faculty of Medicine, Mansoura University, Mansoura 35516, Egypt
5. Dep. of Internal Medicine, Infectious Diseases Div., College of Medicine, University of Cincinnati, Cincinnati, OH 45267, USA
6. Dept. of Agric. Chemistry, Faculty of Agriculture, Mansoura University, Egypt
7. Department of Zoology, Women's College, Ain Shams University, Egypt.
8. Dep. of Molecular Genetics and Biochemistry, College of Medicine, Uni. of Cincinnati, Cincinnati OH, USA
9. Dep. of Clinical Laboratory Sciences, College of Applied Medical Sciences, Taibah University, Madinah, Saudi Arabia
10. Department of Pathology, College of Medicine, Taibah University, Madinah, Saudi Arabia.
11. Department of Pathology and Laboratory Medicine, Ministry of The National Guard-Heath Affairs, Madinah, Saudi Arabia..
12. Department of Microbiology at specialized Medical Hospital, Mansoura University, Mansoura, Egypt.

* Correspondence: e-mail: * alrefahd@ucmail.uc.edu; Tel.: 0015139759195
* m.ab.ibrahim@mu.edu.sa; Tel.: 00966541267818

Table S1. Cytotoxic activity of some compounds against human tumor cells (% inhibition).

Samples	Conc. (μg/mL)	HePG-2	MCF-7	HCT-116	PC3	HeP2	Hela	WI-38
**Doxorubicin**	100	93.7	93.8	92.9	91.2	91.6	92.7	15.2
	50	88.8	89.1	86.1	83.7	84.7	87.9	11.7
	25	85.9	85.7	81.3	78.3	78.3	81.1	9.4
	12.5	71.7	73.1	68.6	61.1	62.1	69.2	6.2
	6.25	54.2	58.5	52.1	40.8	41.8	48.3	3.5
	3.125	42.4	41.6	39.5	26.4	27.4	37.6	2.6
	1.56	28.8	30.9	26.2	4.7	5.7	26	1.4
Ephedra aphylla	100	72.1	67.5	63.6	64.7	72.8	76.4	15.9
	50	61.9	59.3	52.9	50.8	60.2	61.9	15.5
	25	51.8	42.9	35.2	45.2	46.9	50.8	13.6
	12.5	36.3	32	28.1	28.4	38.3	47.3	11.4
	6.25	20.7	10.7	7.7	14.5	12.6	24	8.7
	3.125	7.4	0	0.4	2.7	6.1	4.8	7.8
	1.56	0	0	0	0	0	0	5.6
Ephedra aphylla+ **SeNPs**	100	91.6	79.6	86.8	76.3	84.4	91.7	15.9
	50	85.7	71.9	81.4	68.2	76.1	82.4	14.3
	25	73.9	62.8	72.5	57.4	70.9	74.6	10.6
	12.5	67.1	51.7	63.9	48.1	59.8	61.8	8.3
	6.25	45.8	33.1	37.2	33.5	30.6	42.9	6.8
	3.125	29.2	12.5	20.7	10.6	18.7	22.5	5.1
	1.56	13.5	0	4.6	0	1.5	3.6	3.8
Ephedra aphylla+ **ZnNPs**	100	82.5	79.7	77.2	77.9	83	85.2	15.9
	50	76.1	74.5	70.5	69.2	75.1	76.1	9.7
	25	69.8	64.2	59.4	65.7	66.8	69.2	8.5
	12.5	60.1	57.4	55	55.2	61.4	67	7.1
	6.25	50.4	44.1	42.2	46.5	45.3	52.4	5.5
	3.125	42	37.4	37.6	39.1	41.2	40.4	4.9
	1.56	0	0	0	0	0	0	3.5
**Selenium sulfate**	100	48.5	42.1	45.9	40.4	44.7	48.5	15.9
	50	45.3	38.0	43.1	36.1	40.3	43.6	15.1
	25	39.1	33.2	38.4	30.4	37.5	39.5	13.1
	12.5	35.5	27.4	33.8	25.4	31.6	32.7	11.9
	6.25	24.2	17.5	19.7	17.7	16.2	22.7	11.1
	3.125	15.4	6.6	11.0	5.6	9.9	11.9	10.2
	1.56	7.1	0	2.4	0	0.8	1.9	9.5
**Zinc sulfate**	100	38.1	35.7	33.7	34.2	38.5	40.4	15.9
	50	32.8	31.4	28.0	26.9	31.9	32.8	15.7
	25	27.4	22.7	18.6	23.9	24.8	26.9	14.7
	12.5	19.2	16.9	14.9	15.0	20.3	25.0	13.6
	6.25	11.0	5.7	4.1	7.7	6.7	12.7	12.1
	3.125	3.9	0	0.2	1.4	3.2	2.5	11.7
	1.56	0	0	0	0	0	0	10.5

Table S2. Percent of average relative viability of cells.

Samples	Conc. (µg/ mL)	HePG-2	MCF-7	HCT-116	PC3	HeP2	Hela	WI-38
**Doxorubicin**	100	6.3	6.2	7.1	8.8	8.4	7.3	84.8
	50	11.2	10.9	13.9	16.3	15.3	12.1	88.3
	25	14.1	14.3	18.7	21.7	21.7	18.9	90.6
	12.5	28.3	26.9	31.4	38.9	37.9	30.8	93.8
	6.25	45.8	41.5	47.9	59.2	58.2	51.7	96.5
	3.125	57.6	58.4	60.5	73.6	72.6	62.4	97.4
	1.56	71.2	69.1	73.8	95.3	94.3	74.0	98.6
Ephedra aphylla	100	27.9	32.5	36.4	35.3	27.2	23.6	84.1
	50	38.1	40.7	47.1	49.2	39.8	38.1	84.5
	25	48.2	57.1	64.8	54.8	53.1	49.2	86.4
	12.5	63.7	68	71.9	71.6	61.7	52.7	88.6
	6.25	79.3	89.3	92.3	85.5	87.4	76	91.3
	3.125	92.6	100	99.6	97.3	93.9	95.2	92.2
	1.56	100	100	100	100	100	100	94.4
***Ephedra aphylla*+ SeNPs**	100	8.4	20.4	13.2	23.7	15.6	8.3	84.1
	50	14.3	28.1	18.6	31.8	23.9	17.6	85.7
	25	26.1	37.2	27.5	42.6	29.1	25.4	89.4
	12.5	32.9	48.3	36.1	51.9	40.2	38.2	91.7
	6.25	54.2	66.9	62.8	66.5	69.4	57.1	93.2
	3.125	70.8	87.5	79.3	89.4	81.3	77.5	94.9
	1.56	86.5	100	95.4	100	98.5	96.4	96.2
***Ephedra aphylla*+ ZnNPs**	100	17.5	20.3	22.8	22.1	17	14.8	84.1
	50	23.9	25.5	29.5	30.8	24.9	23.9	90.3
	25	30.2	35.8	40.6	34.3	33.2	30.8	91.5
	12.5	39.9	42.6	45	44.8	38.6	33	92.9
	6.25	49.6	55.9	57.8	53.5	54.7	47.6	94.5
	3.125	58	62.6	62.4	60.9	58.8	59.6	95.1
	1.56	100	100	100	100	100	100	96.5
**Selenium sulfate**	100	51.5	57.9	54.1	59.6	55.3	51.5	84.1
	50	54.7	62.0	56.9	63.9	59.7	56.4	84.9
	25	60.9	66.8	61.6	69.6	62.5	60.5	86.9
	12.5	64.5	72.6	66.2	74.6	68.4	67.3	88.1
	6.25	75.8	82.5	80.3	82.3	83.8	77.3	88.9
	3.125	84.6	93.4	89.0	94.4	90.1	88.1	89.8
	1.56	92.9	100	97.6	100	99.2	98.1	90.5
**Zinc sulfate**	100	61.9	64.3	66.3	65.8	61.5	59.6	84.1
	50	67.2	68.6	72.0	73.1	68.1	67.2	84.3
	25	72.6	77.3	81.4	76.1	75.2	73.1	85.3
	12.5	80.8	83.1	85.1	85.0	79.7	75.0	86.4
	6.25	89.0	94.3	95.9	92.3	93.3	87.3	87.9
	3.125	96.1	100	99.8	98.6	96.8	97.5	88.3
	1.56	100	100	100	100	100	100	89.5
